# Supplementary material for: Reproductive, Obstetric and Neonatal Outcomes in Women with Congenital Uterine Anomalies: A Systematic Review and Meta-Analysis
Source: J Clin Med. 2021 Oct 20;10(21):4797. doi: 10.3390/jcm10214797 (PMC8584292; doi:10.3390/jcm10214797)
Supplement: Supplementary file 1 [file jcm-10-04797-s001.zip › jcm-1397142-supplementary.pdf]

Table S1-Search strategies (Date of search: May 8, 2021)

MEDLINE

Search	Query	Items found
**1**	("Uterus"[Mesh]) AND "Congenital Abnormalities"[Mesh]	3,302
**2**	(unicornuate[tiab] OR didelphys[tiab] OR didelphus[tiab] OR bicornuate[tiab] OR septa*[tiab] OR septu*[tiab] OR subsepta*[tiab] OR subseptu*[tiab] OR sub-septa*[tiab] OR sub-septu*[tiab] OR T-shape*[tiab] OR arcuate[tiab]) AND (uterus[tiab] OR uterine[tiab])	2,974
**3**	(Mullerian anomal*[tiab] OR Mullerian abnomalit*[tiab])	472
**4**	#1 OR #2 OR #3	5,872
**5**	"Pregnancy"[Mesh] OR "Pregnancy Complications"[Mesh]	946,765
**6**	("Delivery, Obstetric"[Mesh] OR "Infant, low birth weight"[Mesh] OR "Apgar Score"[Mesh] OR "Fetal growth retardation"[Mesh] OR "Pregnancy, ectopic"[Mesh] OR "Intensive Care, Neonatal"[Mesh] OR "Reproductive techniques"[Mesh] OR "Infertility, female"[Mesh])	322,265
**7**	((Infant OR perinatal OR fetal OR maternal) AND (death OR compromise OR mortality OR morbidity)) OR ((pregnancy OR reproductive OR neonatal OR maternal) AND (outcome*)) OR ((Premature OR preterm) AND (labor OR delivery OR birth)) OR (Fetal AND (malpresentation OR malposition)) OR ((Pregnancy OR postpartum OR antepartum OR prepartum) AND (bleeding OR hemorrhage)) OR (Placenta* AND (previa OR abrupt*)) OR ((Retain* OR retention) AND placent*) OR ((Embryo OR blastocyst OR oocyte) AND transfer*)	964,037
**8**	APGAR[tiab] OR "Intrauterine growth restriction"[tiab] OR Cesarean[tiab] OR Caesarean[tiab] OR "Obstructed labor"[tiab] OR preeclampsia[tiab] OR pre-eclampsia[tiab] OR miscarriage[tiab] OR Fertility[tiab] OR conception[tiab] OR "Assisted reproduction"[tiab] OR "Assisted reproductive"[tiab] OR Ivf[tiab] OR ICSI[tiab] OR "In vitro fertilization"[tiab] OR "Intracytoplasmic sperm injection"[tiab] OR "small for gestational age"[tiab]	280,043
**9**	#5 OR #6 OR #7 OR #8	1,648,261
**10**	#4 AND #9	3,017

**11**	#10 NOT ("review"[Publication Type] OR "review literature as topic"[MeSH Terms]))	2,643

EMBASE

**Search**	**Query**	**Items found**
**1**	'uterus malformation'/exp	6,849
**2**	(('unicornuate' OR 'didelphys' OR 'didelphus' OR 'bicornuate' OR 'septa*' OR 'septu*' OR 'subsepta*' OR 'subseptu*' OR 'sub-septa*' OR 'sub-septu*' OR 't-shape*' OR 'arcuate') NEAR/2 ('uterus' OR 'uterine')):ab,ti	3,306
**3**	(mullerian NEXT/2 anomal*) OR (mullerian NEXT/2 abnomalit*)	1,232
**4**	#1 OR #2 OR #3	8,635
**5**	'pregnancy'/exp OR 'abortion'/exp OR 'placenta disorder'/exp OR 'pregnancy complication'/exp OR 'labor complication'/exp OR 'ectopic pregnancy'/exp OR 'fetus disease'/exp OR 'high risk pregnancy'/exp OR 'obstetric emergency'/exp OR 'prolonged pregnancy'/exp OR 'postpartum hemorrhage'/exp OR 'uterine atony'/exp OR 'maternal morbidity'/exp OR 'uterus contraction'/exp OR 'uterine cervix ripening'/exp OR 'low birth weight'/exp OR 'prematurity'/exp OR 'immaturity'/exp OR 'infertility therapy'/exp OR 'intrauterine growth retardation'/exp OR 'infertility'/exp	1,264,713
**6**	(infant OR perinatal OR fetal OR maternal) AND (death OR compromise OR mortality OR morbidity) OR ((pregnancy OR reproductive OR neonatal OR maternal) AND outcome*) OR ((premature OR preterm) AND (labor OR delivery OR birth)) OR (fetal AND (malpresentation OR malposition)) OR ((pregnancy OR postpartum OR antepartum OR prepartum) AND (bleeding OR hemorrhage)) OR (placenta* AND (previa OR abrupt*)) OR ((retain* OR retention) AND placent*) OR ((embryo OR blastocyst OR oocyte) AND transfer*)	661,958
**7**	'apgar':ab,ti OR 'cesarean':ab,ti OR 'caesarean':ab,ti OR 'obstructed labor':ab,ti OR 'preeclampsia':ab,ti OR 'pre-eclampsia':ab,ti OR 'miscarriage':ab,ti OR 'fertility':ab,ti OR 'conception':ab,ti OR 'assisted reproduction':ab,ti OR 'assisted reproductive':ab,ti OR 'ivf':ab,ti OR 'icsi':ab,ti	370,781

	OR 'in vitro fertilization':ab,ti OR 'intracytoplasmic sperm injection':ab,ti OR 'iugr':ab,ti OR 'small for gestational age':ab,ti	
**8**	#5 OR #6 OR #7	1,622,469
**9**	#4 AND #8	5,314
**10**	#9 NOT ('animal cell'/de OR 'animal experiment'/de OR 'animal model'/de OR 'animal tissue'/de OR 'nonhuman'/de)	4,463
**11**	#10 NOT 'review'/it	4,088

COCHRANE

**Search**	**Query**	**Items found**
**1**	MeSH descriptor: [Uterus] explode all trees	2,885
**2**	MeSH descriptor: [Congenital Abnormalities] explode all trees	6,231
**3**	#1 and #2	22
**4**	((unicornuate or didelphys or didelphus or bicornuate or septa* or septu* or subsepta* or subseptu* or sub-septa* or sub-septu* or T-shape* or arcuate) and (uterus or uterine or uteri*)):ti,ab,kw (Word variations have been searched)	161
**5**	Mullerian anomal* or Mullerian abnomalit*:ti,ab,kw (Word variations have been searched)	33
**6**	#3 or #4 or #5	206
**7**	MeSH descriptor: [Pregnancy] explode all trees	22,464
**8**	MeSH descriptor: [Pregnancy Complications] explode all trees	12,119
**9**	MeSH descriptor: [Delivery, Obstetric] explode all trees	5,301
**10**	MeSH descriptor: [Infant, Low Birth Weight] explode all trees	2,223
**11**	MeSH descriptor: [Apgar Score] explode all trees	693
**12**	MeSH descriptor: [Fetal Growth Retardation] explode all trees	400
**13**	MeSH descriptor: [Pregnancy, Ectopic] explode all trees	179
**14**	MeSH descriptor: [Intensive Care, Neonatal] explode all trees	335
**15**	MeSH descriptor: [Reproductive Techniques] explode all trees	3,964
**16**	MeSH descriptor: [Infertility] explode all trees	3,459
**17**	((Infant OR perinatal OR fetal OR maternal) AND (death OR compromise OR	71,258

	mortality OR morbidity)) OR ((pregnancy OR reproductive OR neonatal OR maternal) AND (outcome*)) OR ((Premature OR preterm) AND (labor OR delivery OR birth)) OR (Fetal AND (malpresentation OR malposition)) OR ((Pregnancy OR postpartum OR antepartum OR prepartum) AND (bleeding OR hemorrhage)) OR (Placenta* AND (previa OR abrupt*)) OR ((Retain* OR retention) AND placent*) OR ((Embryo OR blastocyst OR oocyte) AND transfer*) (Word variations have been searched)	
**18**	APGAR or "Intrauterine growth restriction" or Cesarean or Caesarean or "Obstructed labor" or preeclampsia or pre-eclampsia or miscarriage or Fertility or conception or "Assisted reproduction" or "Assisted reproductive" or Ivf or ICSI or "In vitro fertilization" or "Intracytoplasmic sperm injection" or "small for gestational age":ti,ab,kw (Word variations have been searched)	63,351
**19**	#7 or #8 or #9 or #10 or #11 or #12 or #13 or #14 or #15 or #16 or #17 or #18	93,471
**20**	#6 and #19	154

Table S2. Study Quality Assessment for Cohort studies (Newcastle-Ottawa Scale)

Study	Selection				Comparability	Outcome			Total Number of Stars
	Exposed Cohort	Non-exposed Cohort	Ascertainment of Exposure	Outcome of Interest		Assessment of Outcome	Length of Follow-Up	Adequacy of Follow-Up	
Forde, 1978	*	*	*	/	/	*	*	*	6
Sorensen and Trauelsen, 1987	*	*	*	*	/	*	*	*	7
Ben-Rafael, 1991	*	*	*	/	/	*	*	*	6
Acien, 1993	*	*	*	*	/	*	*	*	7
Maneschi, 1995	*	*	*	*	/	*	*	*	7
Zupi, 1996	*	*	*	*	/	*	*	*	7
Cooney, 1998	*	*	*	/	*	*	*	*	7
Ravasia, 1999	*	*	*	*	/	*	*	*	7
Woelfer, 2001	*	*	*	*	/	*	*	*	7
Shuiqing, 2002	*	*	*	*	/	*	*	*	7
Erez, 2007	*	*	*	*	/	*	*	*	7
Zlopasa, 2007	*	*	*	*	/	*	*	*	7
Ban-Frangez, 2009	*	*	*	*	**	*	*	*	9
Liang, 2010	*	*	*	/	*	*	*	*	7
Saravelos, 2010	*	*	*	*	/	*	*	*	7
Zhang, 2010	*	*	*	*	/	*	*	*	7
Hua, 2011	*	*	*	/	**	*	*	*	8
Jayaprakasan, 2011	*	*	*	*	*	*	*	*	8
Fox, 2014	*	*	*	/	/	*	*	*	6
Takami, 2014	*	*	*	/	/	*	*	*	6
Hiersch, 2016	*	*	*	/	/	*	*	*	6
Li, 2017	*	*	*	/	/	*	*	*	6
Mastrolia, 2017	*	*	*	*	/	*	*	*	7
Ozugur, 2017	*	*	*	/	*	*	*	*	7
Mastrolia, 2018	*	*	*	*	/	*	*	*	7
Ples, 2018	*	*	*	*	/	*	*	*	7
Prior, 2018	*	*	*	*	/	*	*	*	7
Cahen-Peretz, 2019	*	*	*	*	/	*	*	*	7
Chen, 2019	*	*	*	*	**	*	*	*	9
Neal, 2019	*	*	*	/	/	*	*	*	6
Ouyang, 2020	*	*	*	*	/	*	*	*	7
Kong, 2021	*	*	*	*	**	*	*	*	9
Zambrotta, 2021	*	*	*	/	/	*	*	*	6
Zhang, 2021	*	*	*	*	/	*	*	*	7

Table S3. Study Quality Assessment for Case-Control Studies (Newcastle-Ottawa Scale)

**Study**	**Selection**				**Comparability**	**Exposure**			**Total Number of Stars**
	**Definition of Cases**	**Representativeness of Cases**	**Selection of Controls**	**Definition of Controls**		**Assessment of Exposure**	**Method of Ascertainment**	**Non-Response Rate**	
Sugiura-Ogasawara, 2010	*	*	*	*	/	*	*	/	6
Tomazevic, 2010	*	*	*	*	**	*	*	/	8
Chen, 2018	*	*	*	*	*	*	*	/	7
